# Analysis of the Ordinary and Extraordinary Ionospheric Modes for NVIS Digital Communications Channels

**DOI:** 10.3390/s21062210

**Published:** 2021-03-22

**Authors:** Jordi Male, Joaquim Porte, Tomas Gonzalez, Josep M. Maso, Joan L. Pijoan, David Badia

**Affiliations:** La Salle Campus, Ramon Llull University, 08022 Barcelona, Spain; jordi.male@salle.url.edu (J.M.); joaquim.porte@salle.url.edu (J.P.); tomas.gonzalez@salle.url.edu (T.G.); josep.maso@salle.url.edu (J.M.M.); david.badia@salle.url.edu (D.B.)

**Keywords:** HF, NVIS, SIMO, diversity combining, sounding, communication channel, ionosphere, STANAG, MIL-STD-188, polarization diversity, remote sensing

## Abstract

Sensor networks have become more popular in recent years, now featuring plenty of options and capabilities. Notwithstanding this, remote locations present many difficulties for their study and monitoring. High-frequency (HF) communications are presented as an alternative to satellite communications, being a low-cost and easy-to-deploy solution. Near vertical incidence skywave (NVIS) technology provides a coverage of approximately 250 km (depending on the frequency being used and the ionospheric conditions) without a line of sight using the ionosphere as a communication channel. This paper centers on the study of the ionosphere and its characteristic waves as two independent channels in order to improve any NVIS link, increasing its robustness or decreasing the size of the node antennas through the appliance of specific techniques. We studied the channel sounding of both the ordinary and extraordinary waves and their respective channels, analyzing parameters such as the delay spread and the channel’s availability for each wave. The frequency instability of the hardware used was also measured. Furthermore, the correlation coefficient of the impulse response between both signals was studied. Finally, we applied polarization diversity and two different combining techniques. These measurements were performed on a single frequency link, tuned to 5.4 MHz. An improvement on the mean bit energy-to-noise power spectral density (E_b_/N_0_) was received and the bit error rate (BER) was achieved. The results obtained showed that the extraordinary mode had a higher availability throughout the day (15% more availability), but a delayed spread (approximately 0.3 ms mean value), similar to those of the ordinary wave. Furthermore, an improvement of up to 4 dB was achieved with the usage of polarization diversity, thus reducing transmission errors.

## 1. Introduction

The ionosphere has an essential function for our planet, which is the protection against external radiations. It has been studied for a long time both in a physical way and also as a communication channel [1,2], as the ionosphere behaves like a mirror for high frequency (3–30 MHz) signals. Using the ionosphere as a channel, and taking advantage of the benefits of signal reflection for HF, has wide use for emergency services and is also suitable for ubiquitous sensors networks (USN). In addition, it avoids the use of satellites and high infrastructure and operational costs.

The behavior of the ionosphere is under continuous research due to its difficult prediction. Observatories, using an ionosonde, examine the quantity of ions and electrons produced in the atmosphere to get information of the radio wave refraction, and the generation of different waves due to these reflections (the ordinary and the extraordinary waves [1,2]). There are studies such as those by the respective authors of [3,4,5], where a deterministic model of a narrowband and wideband HF channel was studied giving a prediction of quality parameters of ionospheric communications, but there was no distinction between ordinary and extraordinary waves. Doppler and multipath measurements on oblique and vertical ionospheric paths were performed in studies by the respective authors of [6,7] and also in the Doppler And Multipath Sounding Network (DAMSON) project [8,9]. Moreover, another sounding of narrowband and wideband ionospheric communications, was done with oblique transmissions [10]. It is known that the availability of the ionosphere as a communication channel is not remarkable, since it depends on its ionization. Its behavior varies throughout the day, which implies that different transmission frequencies must be used due to the change of the critical frequency. Due to its low bandwidth and coherence time, the ionosphere is not a solution for high-speed data transmissions but, on the other hand, its channel characteristics are suitable for sensor network deployment.

In this article, we analyze the behavior of the ionosphere as a communication channel, using near vertical incidence skywave (NVIS) propagation and the transmission of different data frames. The transmit frequency used in our experiment was 5.4 MHz. NVIS propagation is based on the transmission of a signal with an incidence angle between 70° and 90° to the ionosphere. The properties of the ionosphere cause that signal to be reflected, obtaining a coverage of approximately a 250 km radius [11], which is a relevant fact for remote sensors or emergency communications for places without infrastructure [12]. The ionospheric reflection is frequency dependent, with typical frequencies ranging from 3 to 10 MHz. The coverage radius of 250 km corresponds with F_2_-layer propagation.

In what follows, characteristics of the ionosphere and NVIS communications and polarization diversity are introduced in Section 2. The sounding system implemented is described in Section 3, where, in Section 3.1, the overall infrastructure is explained, the data frames designed and used are detailed in Section 3.2, and the test scenario is described in Section 3.3. Results of this study are presented in Section 4, and finally, the conclusions of this work are in Section 5.

## 2. The Ionosphere and Polarization Diversity

The ionosphere is one of the layers of the atmosphere that, thanks to its physical characteristics, allows the refraction of radio signals between 3 and 30 MHz. Specifically, the ionization of the ionosphere is the responsible of this signal refraction. The ionization of the outer layers of the atmosphere depends on the degree of solar activity, which follows cycles of approximately 11 years and presents sunspots as an indicator [1]. The condition of the ionosphere not only changes annually, but also depends on the season and the time of day. These variations make ionospheric communications very challenging, requiring a system that adapts to the state of the ionosphere at all times.

**Figure 1:**
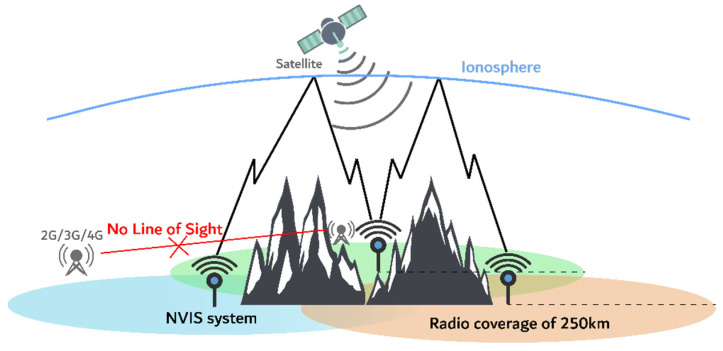
Near vertical incidence skywave (NVIS) link scenario.

Communications through the ionosphere are classified according to the angle of incidence of the radio wave. NVIS communications are based on a 90° to 70° angle of incidence, and generate a coverage area of up to 250 km (depending on frequency and ionospheric conditions) from the point of transmission [11]. The focus of this article is to define the physical properties of the NVIS channel (Figure 1).

The ionosphere presents multiple layers (D layer, E layer, and F layer, which splits into F_1_ and F_2_ layers during daytime), which depend on the sun’s ionization [1]. NVIS communications can use both the E layer and the F_2_ layer. Taking into account the distance between our nodes, and in order to maximize the received signal strength, we based our study on the F_2_-layer propagation. Furthermore, the ionosphere is a birefringent medium. Two modes or propagation (ordinary and extraordinary) are formed as soon as the radio wave enters the ionized plasma in the presence of a magnetic field. The plane-polarized wave is decomposed into two different waves, and the direction of energy is deviated from the direction of propagation [1]. This partition creates two totally different propagation paths, resulting in two independent communication channels.

Specifically, when a radio wave reaches the ionosphere, the electrons in the layer start an elliptical movement [1]. As a result of this almost-circular spin, the radio wave has its polarization changed by the ionosphere. This leads to the return to the Earth of two different rays (the ordinary and extraordinary rays) with different properties, such as different critical frequencies, phase, amplitude, and arrival time [2]. Specifically, both of these waves have elliptical polarization and also have opposite rotation sense. For the Northern Hemisphere, the ordinary wave has the greater delay and left-hand circular polarization (LHCP), and the extraordinary wave presents the lesser delay and right-hand circular polarization (RHCP) [13]. These different properties can be used to improve telecommunication links, as polarization diversity techniques are an option in ionospheric channels.

The different polarizations and the usage of the ionospheric characteristic waves as two different communication channels allow for the usage of polarization diversity techniques to improve the robustness and throughput of the link. The concept of polarization diversity was first introduced by the authors of [14] in the 1950s. The work presented in Reference [15] was one of the first to use polarization diversity at the receiver achieving 9600 bps for a 1800 km skywave link. The authors of [16] highlighted the importance of using both ordinary and extraordinary waves for multiple-input multiple-output (MIMO) in the case of NVIS propagation, and the cross-correlation of both channels was analyzed for narrowband transmissions. A channel model for dual polarized MIMO communications was proposed in [17] and some high throughput testbeds are presented in [18,19], where the improved channel capacity was analyzed. Our team, after evaluating the polarization diversity for long-haul HF links between the Antarctic and Spain [20], is now considering the dual-polarized reception for a NVIS sensors network in order to decrease either the transmission power or the size of the antennas.

In order to improve the robustness of the NVIS link and apply polarization diversity techniques, a combination of the different signals that arrive at the receiver is needed. There are multiple methods of diversity combining, each one presenting different characteristics and gains. We studied two different techniques: equal-gain combining, a method that sums all the received signals coherently, and selection combining, a technique that selects the strongest signal received (a higher signal-to-noise ratio (SNR)) and ignores the other.

## 3. Sounding System

All the hardware used in order to carry out this work is presented in this section. Firstly, a description of the overall system is presented, explaining all the infrastructure and peripherals used. Secondly, the transmitted data frames are listed and detailed. Finally, the implemented link and the realized tests are described.

### 3.1. System Description

**Figure 2:**
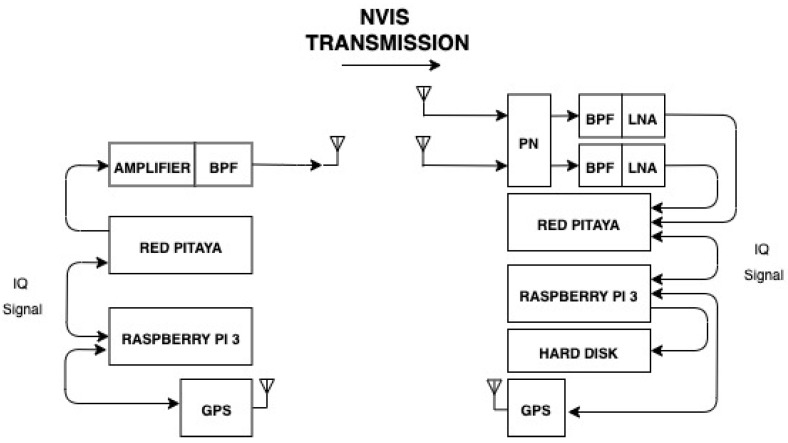
System block diagram.

**Figure 3:**
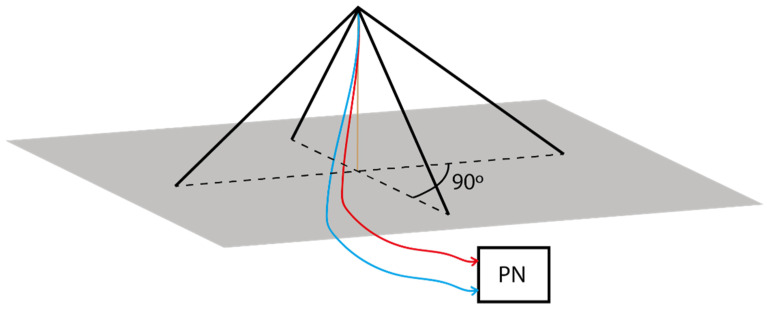
Diagram of the orthogonal inverted vee antennas in the receiver.

**Figure 4:**
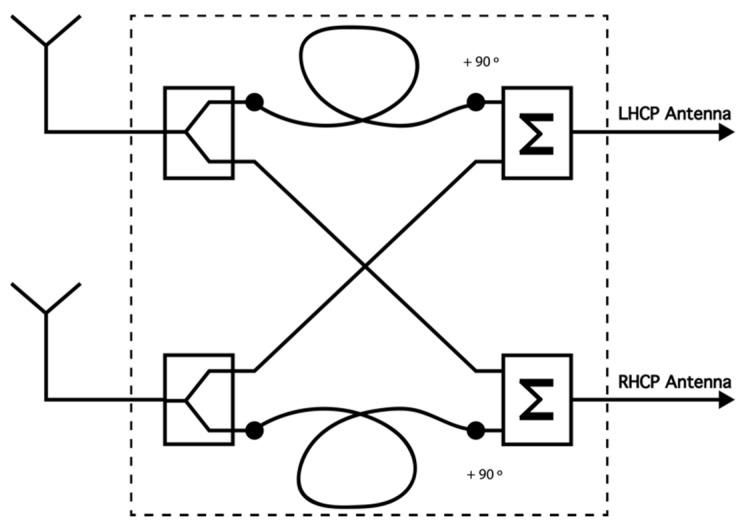
Block diagram of the phasing network.

The system to perform this study relies on a software defined radio (SDR), which can be seen in Figure 2. The versatility of the SDR offers the possibility to adjust parameters for the adaptation of different scenarios. Our SDR was implemented with a field-programmable gate array (FPGA) combined with the Zynq-7010-SOC [21], which were placed in a Red Pitaya STEMlab 125-14 board [22]. This board makes all the computing operations possible, since it features analog-to-digital converters (ADC) and digital-to-analog converters (DAC) with a resolution of 14 bits, all driven by a system clock of 125 MSPS. The Red Pitaya board was connected via Ethernet to a Raspberry Pi 3 [23], which saved the received files onto a hard disk and managed the different peripherals [24]. These connections are presented below:

- Antennas: At the transmitter site, an inverted vee (V-) antenna was used, which was placed in La Salle URL in Barcelona. At the receiver side in Cambrils, two orthogonal inverted-V antennas were located. Figure 3 displays a graphical representation of the orthogonal antennas located in Cambrils. The frequency is currently set to 5.4 MHz, a value based on ionogram studies [25]. The height of the antennas is 14.5 m and the length of their legs is 14 m.
- Phasing Network: The two perpendicular inverted vee antennas worked together with a phasing network (PN; in Figure 2), which was in charge of shifting the phase of one of the two receiver antennas to make it possible to receive different and orthogonal polarizations [26]. The phasing network got a total of four wires, two from each antenna, as we duplicated the received signals using a radio frequency splitter (PDML-20A-100 from Merrimac Industries, Inc.). The route that both antennas followed was the same: one cable was lengthened with a quarter-wave phasing line to provide a 90° shift and connected to a radio frequency (RF) combiner (PDML-20A-100 from Merrimac Industries, Inc.), and the other feed line was directly connected to a RF combiner. The output of the PN gave us a phase difference between the inverted vee antennas of either +90° or −90°. A block diagram of the phasing network is displayed in Figure 4.
- Amplifier: In order to do the sounding an A-class amplifier was used in the transmitter side. The model chosen was the Bonn BLWA 0103-250, which achieves 250 W of maximum power with an input power of 0 dBm.
- Low-noise amplifier (LNA): The model chosen was the ZFL-500LN+, with a minimum gain of 20 dB, a frequency range between 0.05 and 500 MHz, and an operating temperature between −20 °C and 70 °C.
- Filters: We used two band-pass filters (BPF) to avoid known interferences on both sides. On the transmitter side, we filtered the NVIS useful frequency range from 3 to 7 MHz. On the other side, we used a filter with a band pass between 4 and 6 MHz. Our system compensated for the phase delay of the BPF via software, as each data frame was corrected in both amplitude and phase before being demodulated and studied.
- GPS: A GPS was used to synchronize the transmitter and receiver in time (fundamental for the channel study performed). Time synchronization is essential to automate tests and data analysis. Our experiment had different signals sent, which depend on the minute of transmission. Thanks to the time synchronization, the transmitter knows which data file to send and the receiver tags it before saving it in order to analyze the data correctly. The transmitter and receiver were configured with extreme precision thanks to the GPS modules incorporated into the Raspberry. Furthermore, we also used PN sequences to detect the start of the received data structures and synchronize the transmitter and the receiver.

### 3.2. Data Frame Design

**Figure 5:**
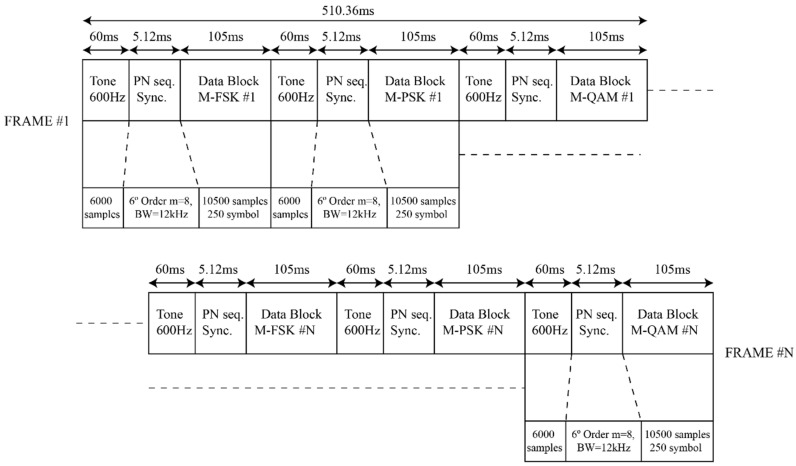
Data frame design [27].

The correct definition of the data frame was essential for the experiment. We defined a level one frame of the Open Systems Interconnection model (OSI model), that consists of a physical structure of the transmitted data. We named this level one frame the “data frame”. A poor definition of the data frame could imply intersymbol interference (ISI) and signal-to-noise ratio (SNR) fadings. Two different data frames were used to perform our tests, which were designed on the basis of earlier studies and the soundings of the ionospheric channel [27]. Figure 5 displays a graphical representation of the first type of signal sent (Frame number 1), which was composed by a total of 50 data groups (we named these structures “packets”), each formed by three different modulations: Phase-shift keying (PSK), frequency-shift keying (FSK), and quadrature amplitude modulation (QAM). All the packets added a preamble that aims to mitigate the negative effects of the ionospheric channel and the frequency deviation between the transmitter and the receiver (the preamble is used to analyze and compensate the received signal’s phase and amplitude via software). This preamble consisted of a 600 Hz tone and a sixth-order PN sequence, and it was located at the beginning of each of the 50 transmitted packets. The sampling speed of the system was 100 kS/s.

Figure 5 exhibits the data frame’s duration at both the sample and time levels. Analyzing all the packet segments and their respective lengths, it can be observed that the 600 Hz tone’s duration was 6000 samples, the PN’s sequence duration was 512 samples, and the modulated data transmitted corresponded to 10,500 samples. Furthermore, every data block contained 250 symbols with a resample of 42 (10,500 samples divided by 250 symbols), resulting in a bandwidth of 2.38 kHz per data block. On the other hand, the bandwidth used in the PNs was 12 kHz. This is because our frame had to respect the coherence time of the ionospheric channel, and in the design of the data frame, we did not want the PN sequences to have a significant influence. Our team decided to make the pseudo-random sequences shorter in time, resulting in a bandwidth of 12 kHz.

The data frame designed had a total duration of 510.36 ms, which was less than the most restrictive coherence time of the ionospheric channel (1.46 s) [28]. The total duration of the 50 packets sent was 25.518 s.

The first tone of the data frame was preceded by an extra block made of a PN sequence, intended to synchronize the system sample-wise.

All the data frames received were stored to be treated afterwards. The processing applied to each one of the data frames is explained as follows: First of all, the system identifies the data frame by correlating the signal received with the value of the PN sequence transmitted. If there are equispaced peaks in the result of this correlation, a data frame is identified. Once the system identifies the data frame, the first block encountered is the 600 Hz tone. This tone of a duration of 60 ms is key to identify and correct the channel’s Doppler shift. In our system, the Doppler shift could not be studied as a frequency offset, as the Red Pitaya platform clocks have a low stability and create a relative frequency-drift effect that is higher than the ionospheric channel. Measures of the platform show that the maximum value of the frequency offset received, due to the low stability clocks, is about ±20 Hz [29]. The 600 Hz tone added to the data frame helps in identifying the frequency instability inserted by its variations between 580 and 620 Hz, approximately. Once the frequency offset is calculated, the received signal is corrected and this frequency offset is compensated for a correct demodulation of the signal.

The second block included in the data frame’s preamble is the sixth-order PN sequence, whose function is to identify the start of the modulated data. The resampling of the PN sequence was about eight and had a total duration of 5.12 ms, as shown in Figure 5. The modulated data blocks were located just after the PN sequence.

**Figure 6:**
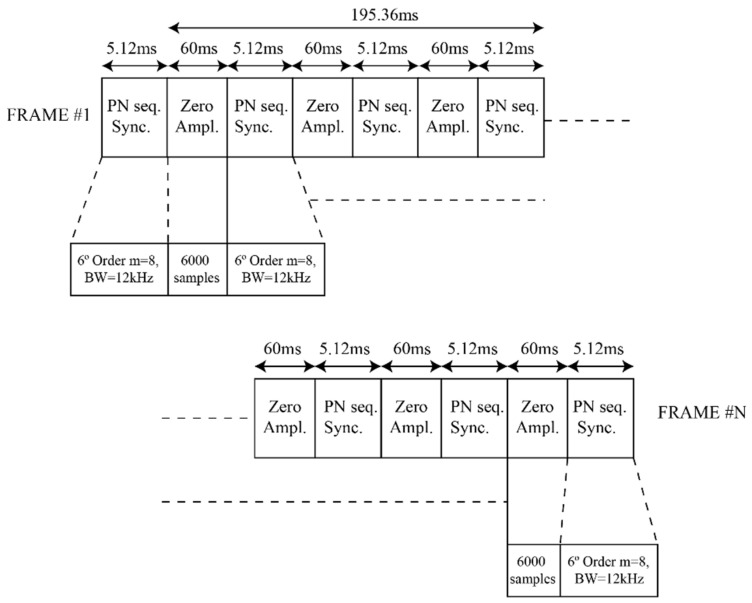
Second data frame design.

As shown in Figure 6, a second data frame was designed for the study of the correlation coefficient between the ordinary and extraordinary channels and their respective delay spread. This second data frame consists of a group of equispaced PN sequences. The spaces between the known sequences do not present any kind of signal, and present a theoretical value of zero amplitude. The purpose of this design is the correct correlation of the PN sequences, as it is fundamental for the correct computation of the multipath values and the correlation coefficients between channels.

A key factor for our study was the separation between PN sequences, as it indicates the separation between the packets. This separation is the direct measurement cadence of our system. For the first data frame (Figure 5), a measurement was performed every 165 ms (6.06 Hz). For the second data frame used (Figure 6), a measurement was performed every 60 ms (16.17 Hz).

### 3.3. Test Scenario

**Figure 7:**
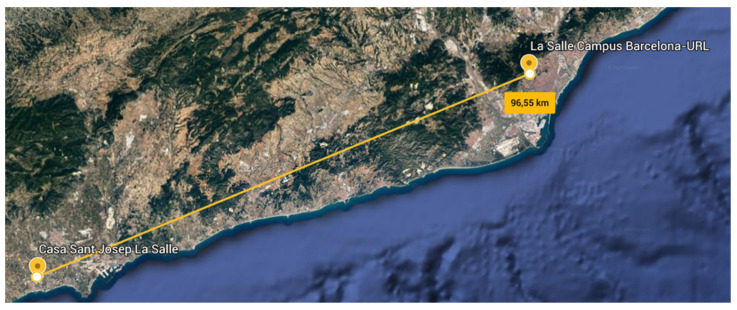
Location of the transmitter and receiver for the channel sounding.

**Figure 8:**
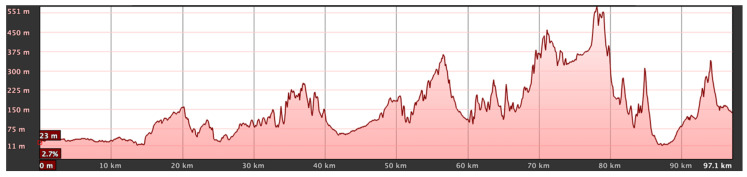
Elevation profile of the NVIS link.

To study the ionospheric channel, the research group installed a sounding system [29] between two points in the Catalonia region (Spain). These NVIS nodes established a link between La Salle University-URL Campus in Barcelona (41.41° N, 2.13° E) and a remote location in Cambrils, Tarragona (41.08° N, 1.07° E). Figure 7 presents a satellite picture of the terrain with the node locations highlighted in yellow. The distance between the two points without line of sight (LOS) was approximately 97 km, a value that is perfectly within the coverage area of an NVIS link. Surface wave signals did not affect our link thanks to the radiation pattern of antennas used in the experiment (their main beam is completely vertical, towards the sky) and the large distance between both points. This was verified as we transmitted different data frames throughout the whole day. During night, when there is absence of ionospheric propagation at 5.4 MHz, we did not receive any signal in our receiver, thus confirming that surface waves do not affect our experiment. Figure 8 presents the elevation profile between the nodes.

Because of the high interferences and electromagnetic noise in the HF band in Barcelona and its surroundings, the receiver was established in Cambrils. This configuration minimized the interferences in the receiving node, thus maximizing the robustness of the link.

The channel study presents a sounding of 12 complete days in December 2019. A total of 8308 files of 29.7 MB were studied, resulting in more than 240 GB of collected data. In one hour, a total of 30 tests were performed. The tests are the transmissions made on our link. These tests follow the format indicated by the experiment, which indicates the data structure to be sent, the transmission power, and the order of modulation sent. These tests followed two different experiments depending on which data frame they were transmitting (Figure 5 or Figure 6). As it can be observed in Table 1, the first data frame transmitted did a transmitting power sweep for five different modulation orders. This experiment is used to evaluate the SDR’s frequency instability by computing the frequency shift of the signal received.

On the other hand, the second data frame (Table 2) did not present any modulated data (no modulation order implied) and was only transmitted at one transmitting power value. This second data frame and its experiment are used to compute the correlation coefficient between both channels (correlation of the impulse responses of both channels), the availability (data frames detected throughout a day), and the delay spread (received multipath of the signal).

## 4. Ionospheric Channels Analysis

This section presents the detailed results obtained with the channel sounding performed. The availability of the ordinary and extraordinary NVIS Channels, their cross-correlation coefficient, and the delay spread are exhibited. Furthermore, the frequency offset caused by the SDR’s frequency difference was also computed. The first three parameters (availability, correlation, and delay spread) were studied and computed using the second data frame (Figure 6), as they focus on data frame detection and PN sequence correlations. The remaining frequency instability was analyzed using the first data frame (Figure 5) as it was computed packet-wise. Finally, we analyzed the usage of polarizations diversity (the combining of the ordinary (O) and extraordinary (X) channels) and its improvement to the robustness of the NVIS link. Two different combining methods were used and studied: selection combining and equal-gain combining. All the results of this study are the product of observing the data sent over the 12 days. The data of all the days was put together in different graphs to analyze the behavior of our link in the described period.

**Figure 9:**
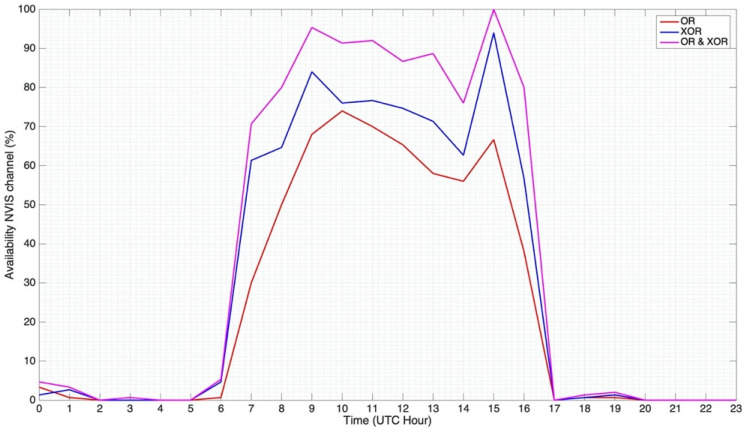
Availability of the Barcelona—Cambrils NVIS ionospheric channels.

First of all, the availability of the ordinary and extraordinary wave’s channels was evaluated. Figure 9 displays the percentage of data frames detected at 5.4 MHz at the reception point in Cambrils. This data frame detection was based on the PN sequences received and their correlation with our known sequence. The availability was defined as follows: the total number of data frames received with respect to the total number of sent data frames. The maximum availability (number of transmitted data frames) was defined as the peak performance (corresponding to the 100% in our graph). The number of data frames detected for every hour was based on this factor and then displayed in the graph. Figure 9 states that the ionospheric channel is not active up until 7 Coordinated Universal Time (UTC) (8 a.m. Central European Time (CET)) and stops being active at 17 UTC time (6 p.m. CET). This result matches with the sunrise and the sunset in the month when the tests were performed (November/December), as it corresponds approximately to the hours of the activation and deactivation of the ionospheric channels. The best availability was between 7 UTC and 16 UTC (8 a.m. and 5 p.m. CET). This high availability corresponds to the day’s highest amount of solar activity. Comparing the ordinary and extraordinary channels, it can be affirmed that the extraordinary channel clearly performed better. The extraordinary wave received reached a peak performance of data frames detected at 15 UTC. Two exceptional intervals (7 UTC and 16 UTC) can be identified in Figure 9, in which almost only the extraordinary wave propagated and right-hand circular polarization (RHCP) was received. The ordinary wave (LHCP) rarely propagated, resulting in availabilities between 30% and 40%. These intervals are known as “happy hours” [13]. At sunrise the ionization showed a steep gradient and, accordingly, the morning happy hour was short (typically 30 min at mid-latitudes in winter, our scenario). The evening happy hour often lasted more than an hour due to the slower ion recombination processes [13]. Consequently, the highest differences in availability between the two channels coincided with the happy hours mentioned. Figure 9 also exhibits the performance (percentage of data frames detected) of both the ionospheric channels. The legend of the graph is defined as follows: OR is the performance of the ordinary wave, XOR is the performance of the extraordinary wave received, and OR and XOR refers to the total performance between both ordinary and extraordinary modes. The results are clearly better, achieving a result of 86% of the data frames detected from 7 UTC to 16 UTC.

The usage of both ionospheric channels at the same time resulted in the reception of simultaneous signals. If these signals are decorrelated (two channels are considered as not correlated when their cross-correlation coefficient value is lower than 0.7 [30]), an increase of SNR (signal-to-noise ratio) can be achieved. MIMO and single-input multiple-output (SIMO) links can benefit directly from this SNR gain, enabling link enhancement by the application of diversity techniques.

**Figure 10:**
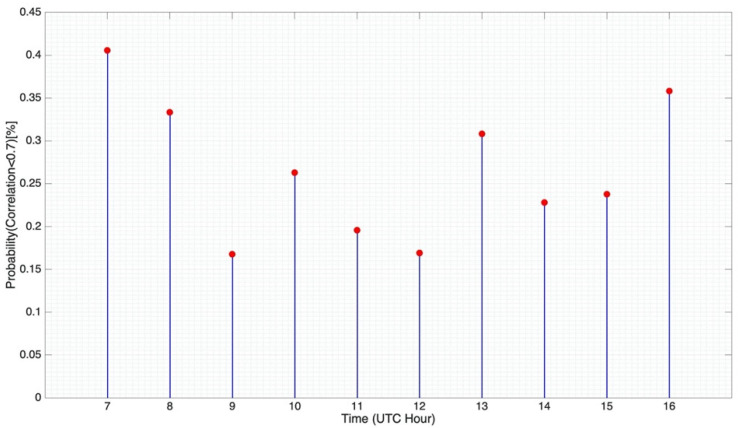
Probability (in %) of finding a cross-correlation coefficient below 0.7 between both channels.

Figure 10 displays the cross-correlation coefficient between the ordinary wave’s channel and the extraordinary wave’s channel throughout the day. This value was computed by correlating the impulse responses of both channels. Before calculating the coefficient, the impulse responses were previously synchronized, so the delay between the received waves was not taken into account. A probability graph was exhibited in order to evaluate if both received signals were decorrelated enough, depending on the hour of the day. Analyzing the results, it can be stated that there is a probability of nearly 40% to achieve a correlation coefficient below 0.7 in the happy hour intervals. The SNR of the received signals in these intervals could be improved by the usage of diversity techniques.

**Figure 11:**
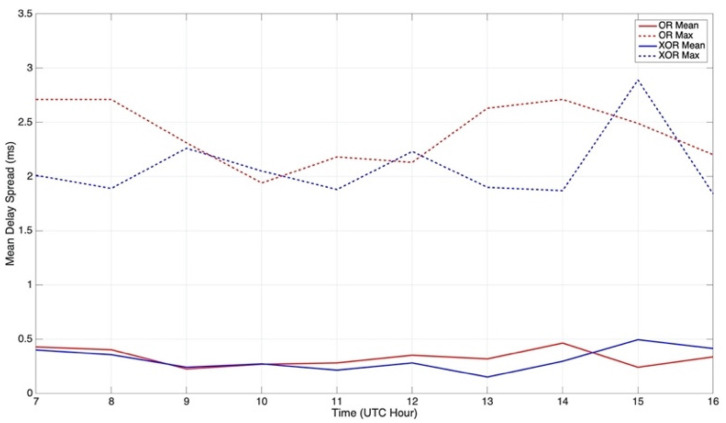
NVIS delay spread vs. time of the NVIS channels.

In Figure 11, the delay spread of the ordinary and extraordinary waves can be analyzed, respectively. The multipath of the NVIS link between Barcelona and Cambrils was studied throughout the day. All the undesired paths limited our channel’s coherence bandwidth, thus affecting the data frame design and the link’s performance.

The figure displayed below only takes into account the well-demodulated data frames. If a data frame did not present enough SNR and the PN sequences were not correctly found, no multipath was computed. Therefore, the following graph was analyzed together with the channel’s availability, presented above (Figure 9). Only the hours where both channels were active (7 UTC to 16 UTC) were taken into account for the delay spread study in order to have accurate results.

**Figure 12:**
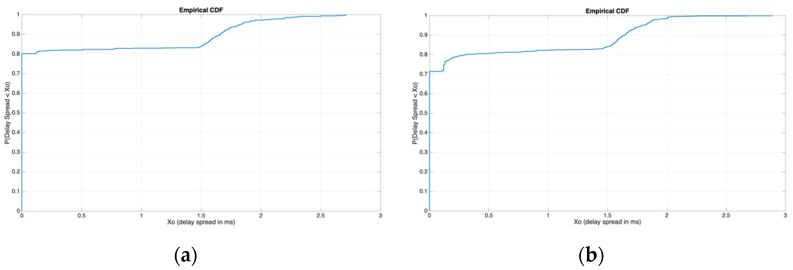
(**a**) NVIS delay spread distribution of the ordinary channel; (**b**) NVIS delay spread distribution of the extraordinary channel.

After carefully analyzing the delay spread of both channels, the highest value of the link was found to be 2.89 ms, corresponding to a coherence bandwidth of 346 Hz, and was provided by the extraordinary wave. On the other hand, the ordinary wave presented a peak value of 2.71 ms, corresponding to 369 Hz of coherence bandwidth. Both values implied receiving strong ISI among symbols in our system if we considered a time symbol of 0.42 ms (standards STANAG and MIL-STD-188 110, 2.38 kHz bandwidth). The coherence bandwidth of the ionospheric channel was thus defined as follows:

CB = 1/σ,(1)

where σ corresponds to the delay spread. If we now study the less restrictive values, it can be observed that both waves often presented no multipath at all, as was observed in the mean values of the delay spread received (very low delay spread values). Therefore, the study of the mean value of the delay spread is key in our system design. The overall mean values of the delay spread of both channels were similar, presenting some differences if the graph was analyzed hour by hour. The differences in the multipath detected could only be observed if we compared all data frames individually, resulting in different instantaneous values. The mean value received of the ordinary wave was 0.33 ms, and the mean value received of the extraordinary wave was 0.31 ms. Taking into account the most-restrictive mean value (0.33 ms), which corresponds to a coherence bandwidth of 3 kHz (higher than our used bandwidth; thus, our system overcame the ISI of the channel in almost all transmissions). Figure 12 displays the distribution of the delay spread of both ionospheric modes independently.

Another fundamental parameter to study for our NVIS system was the frequency offset that our link was affected by. This frequency variation depends on the movement between the transmitter and the receiver, which in our scenario should be produced by the displacement of the physical layers of the ionosphere. Notwithstanding this, the doppler shift caused by the ionosphere was negligible compared to the frequency variation that the clocks of the Red Pitaya platform produced. This shift was directly related to the temperature of the platform, which affected the clock’s stability. The channel study performed in this research was implemented with rather cheap nodes, in a system where low-stability clocks are usual. Accordingly, a good data frame design and a good post-processing of the signals received was key to mitigate the negative effects of the usage of low-cost technologies.

**Figure 13:**
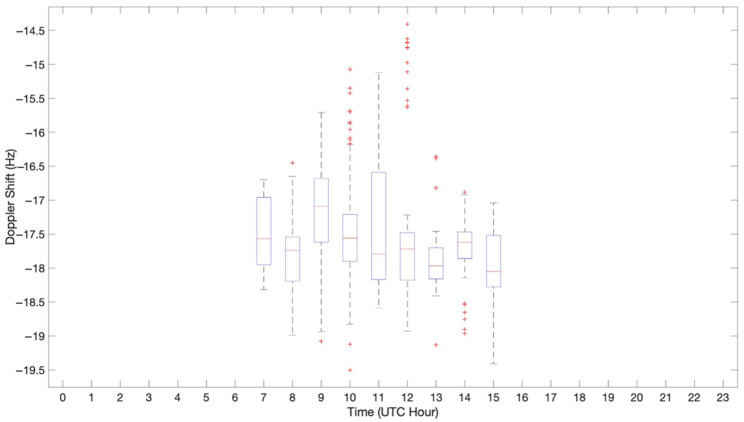
Frequency offset received vs. time.

Figure 13 exhibits a boxplot for all the hours throughout the day when the ionospheric channels are active. It can be appreciated that the maximum frequency offset received was −19.5 Hz and the minimum was −15 Hz, values that were remarkably higher than the ionospheric layer’s Doppler shift (a value that can reach a maximum of 4 Hz, approximately [31]).

Finally, we present the study of the combination of the ordinary and extraordinary wave’s received. Tests on the bit error rate (BER) and bit energy-to-noise power spectral density (E_b_/N_0_) of the system were performed to evaluate the improvement of the robustness achieved. The results of the fourth-order modulations with a transmit power of 50 W are presented below.

First of all, we measured the relationship between the E_b_/N_0_ received from the individual characteristic waves (ordinary and extraordinary) compared to the E_b_/N_0_ received as a result of the application of diversity combining techniques (selection combining and equal-gain combining). The E_b_/N_0_ of the signal received was computed as follows:

E_b_/N_0_ (dB) = SNR(dB) + 10·log 10(B) − 10·log 10(Rb), (2)

where Rb is the signal’s bitrate (depends on the modulation order under test), B is the noise bandwidth of the measurement (2.3 kHz in our scenario), and SNR is the signal-to-noise ratio of the received signal.

**Figure 14:**
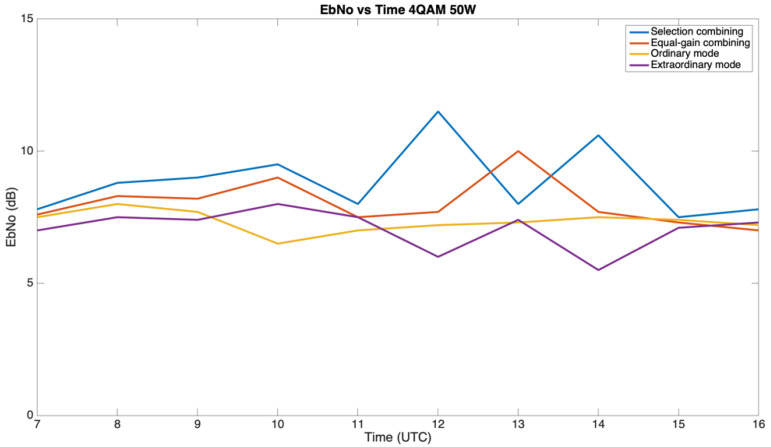
Received bit energy-to-noise power spectral density (E_b_/N_0_) for the 4QAM modulation at 50 W throughout the day.

**Figure 15:**
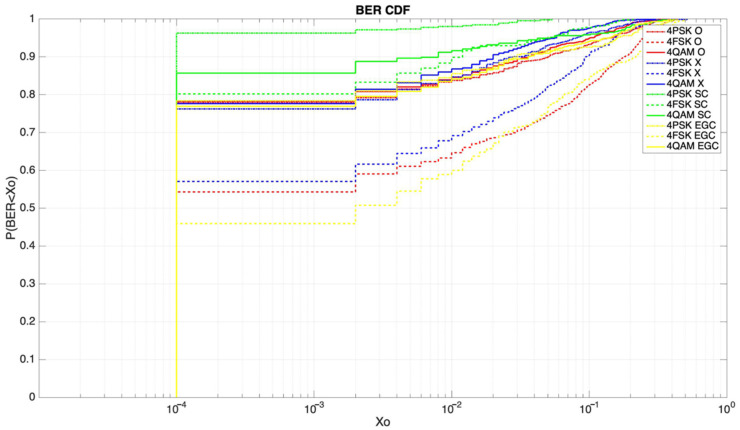
Cumulative distribution function (CDF) of the bit error rate for fourth-order modulations at 50 W.

Figure 14 presents the behavior of the ionospheric waves and their combining by the mean E_b_/N_0_ received throughout the day. Figure 14 only exhibits the 4QAM results in order to make the graph clearer. The 4PSK modulation presented almost identical performances in terms of E_b_/N_0_, while the 4FSK presented worse results. All three modulations are studied in terms of the BER in Figure 15. We can state that the selection combining (SC) technique presented a higher E_b_/N_0_ value than the individual ionospheric waves in all the studied hours. An improvement of up to 4 dB was achieved (at 12 UTC) while using this method. The equal-gain combining (EGC) technique also improved the performance of the link. An improvement of up to 3 dB was achieved (at 13 UTC), but there were certain times of the day (11 UTC, 14–16 UTC) when this technique did not improve the robustness of the link. Finally, we can also see that the mean E_b_/N_0_ received by the O and X modes differed by a maximum of 2 dB. Figure 14 compares the results obtained by using selection combining, equal-gain combining, the ordinary mode, and the extraordinary mode.

Figure 15 presents the BER study performed in our link, specifically the fourth-order modulations with a transmitting power of 50 W. A clear improvement can be observed if we compare the characteristic waves individually with the combining of these techniques. The O and X (ordinary and extraordinary) modes had a 75% to 80% probability to achieve a BER lower than 10^−4^ when using the 4PSK and 4QAM modulations, respectively. If we used selection combining (SC), this probability improved up to 96% and 85%, respectively. On the other hand, if we used equal-gain combining (EGC), the probabilities to receive a BER lower than 10^−4^ improved to 82% for both modulations. The 4FSK modulation was the modulation with the worst performance. For the O and X waves, the 4FSK had a 55% to 57% of probability to achieve a BER lower than 10^−4^, respectively. Using diversity combining, this probability improved up to 88% with SC and 59% with EGC.

## 5. Conclusions

In this work we present the single-frequency study of the two ionospheric characteristic waves as different communication channels. We analyzed the cross-correlation coefficient and the availability of both waves using nearly orthogonal polarized antennas. Similarly, we also studied the delay spread of both channels. We measured the frequency offset caused by the use of low-stability clocks on a low-cost system. Finally, we studied the BER and E_b_/N_0_ improvement of the system with the application of polarization diversity techniques, using selection combining and equal-gain combining. The research carried out in this work focused on two main objectives: the exploration of the feasibility of using polarization diversity techniques thanks to the decorrelation between the ordinary and extraordinary waves, and the study of the properties of each ionospheric channel for the optimization of the data frames in future studies and further channel characterization. From 17 UTC to 6 UTC, the lowest cross-correlation coefficient was found.

This work presents the comparison between all the parameters studied. Table 3 is exhibited below for a better understanding of these parameters.

The availability results showed that the extraordinary wave presented better results. Two “happy hour” [13] intervals were identified (sunrise and evening beginning), where the number of data frames detected by each ionospheric channel differed more. Another study realized in this work analyzed the usage of both signals simultaneously and the data frame detection between them both working together. The mean value of this new availability (from 7 UTC to 16 UTC) was 86%, increasing the results obtained by a singular characteristic wave by more than 13%. These results encourage the study and application of diversity techniques and their combining at the receiver to increase the SNR of this NVIS link.

The delay spread (directly related to the multipath) of both channels was almost identical, a fact that allowed us to use the same coherence bandwidth for both channels. Having a huge value difference between both channels would imply the usage of the most restrictive data frames for the channel with the lower delay spread, not taking full advantage of the parameters of one channel.

The study of the Doppler shift was not performed properly because the system used a low-cost platform (higher frequency offset from the Red Pitaya clocks than the ionosphere’s Doppler shift). Thus, well-designed data frame working together with a powerful post-processing of the signal are key to mitigate the channel’s negative effect (up to ±20 Hz in this work’s infrastructure).

After analyzing the results obtained in the BER and E_b_/N_0_ studies, we can conclude that the application of polarization diversity implies an improvement in the robustness of the link. A higher E_b_/N_0_ and a lower BER were received using both selection combining and equal-gain combining. Selection combining presented the best results, improving the mean E_b_/N_0_ up to 4 dB compared to an individual ionospheric mode, and also remarkably lowering the BER results for the different modulations studied (4PSK, 4FSK, and 4QAM). Furthermore, we can also affirm that the ordinary and extraordinary waves were received with different mean E_b_/N_0_ values, which differed up to 2 dB.

According to our knowledge, no similar studies were performed for NVIS transmissions. The closest study for a channel like ours and the analysis of the different modes of the ionosphere was performed in [28] (a multifrequency study from 2012). Similar results were obtained in the multipath study for both the O and X modes. Our study, in addition, presented the availability of the different modes for NVIS transmissions and their correlation coefficients. The authors of [28] also presented a study of the BER improvement (up to 8% improvement) by the use of polarization diversity. Our system, on the other hand, improved the robustness of NVIS communications much more, up to 33% (4FSK at 50 W).

## Figures and Tables

**Table 1:** Experiment for the first data frame (see Figure 5).

Modulation Order	Transmitting Power	Min
2, 4, 8, 16, 32	3 W	05, 06, 07, 08, 09
2, 4, 8, 16, 32	6 W	15, 16, 17, 18, 19
2, 4, 8, 16, 32	12 W	25, 26, 27, 28, 29
2, 4, 8, 16, 32	25 W	35, 36, 37, 38, 39
2, 4, 8, 16, 32	50 W	45, 46, 47, 48, 49
2, 4, 8, 16, 32	100 W	55, 56, 57, 58, 59

**Table 2:** Experiment for the second data frame (see Figure 6).

Transmitting Power	Min
50 W	05, 06, 07, 08, 09
	15, 16, 17, 18, 19
	25, 26, 27, 28, 29
	35, 36, 37, 38, 39
	45, 46, 47, 48, 49
	55, 56, 57, 58, 59

**Table 3:** Channel study summary.

Parameter	Ordinary Wave			Extraordinary Wave		
	Max	Min	Mean	Max	Min	Mean
Availability (7 UTC to 16 UTC)	74%	30%	57.6%	94%	56.67%	72.2%
Delay Spread	2.71 ms	~0 ms	0.33 ms	2.89 ms	~0 ms	0.31 ms
SDR Frequency offset	−19.5 Hz	−14.5 Hz	−17.7 Hz	−19.5 Hz	−14.5 Hz	−17.7 Hz

## Data Availability

Not applicable.
